# Nomograms for predicting survival in patients with metastatic gastric adenocarcinoma who undergo palliative gastrectomy

**DOI:** 10.1186/s12885-019-6075-5

**Published:** 2019-08-28

**Authors:** Tai Ma, Zhi-jun Wu, Hui Xu, Chang-hao Wu, Jing Xu, Wan-ren Peng, Lu-lu Fan, Guo-ping Sun

**Affiliations:** 1 Department of Oncology, The First Affiliated Hospital of Anhui Medical University, 218 Jixi Road, Hefei, 230022 Anhui Province China; 2 Anhui Institute for Cancer Prevention and Control, 218 Jixi Road, Hefei, 230022 Anhui Province China; 3 Faculty of Health and Medical Sciences, University of Surrey, Guildford, UK

**Keywords:** Gastric adenocarcinoma, Palliative gastrectomy, Nomogram, Prognosis

## Abstract

**Background:**

Recently, evidence has emerged that palliative gastrectomy in patients with stage IV gastric cancer may offer some survival benefits. However, the decision whether to perform primary tumor surgery remains challenging for surgeons, and investigations into models that are predictive of prognosis are scarce. Current study aimed to develop and validate prognostic nomograms for patients with metastatic gastric adenocarcinoma treated with palliative gastrectomy.

**Methods:**

The development dataset comprised 1186 patients from the Surveillance, Epidemiology, and End Results Program who were diagnosed with metastatic gastric adenocarcinoma in 2004–2011, while the validation dataset included 407 patients diagnosed in 2012–2015. Variables were incorporated into a Cox proportional hazards model to identify independent risk factors for survival. Both pre- and postoperative nomograms for predicting 1- or 2-year survival probabilities were constructed using the development dataset. The concordance index (c-index) and calibration curves were plotted to determine the accuracy of the nomogram models. Finally, the cut-off value of the calculated total scores based on preoperative nomograms was set and validated by comparing survival with contemporary cases without primary tumor surgery.

**Results:**

Age, tumor size, location, grade, T stage, N stage, metastatic site, scope of gastrectomy, number of examined lymph node(s), chemotherapy and radiotherapy were risk factors of survival and were included as variables in the postoperative nomogram; the c-indices of the development and validation datasets were 0.701 (95% confidence interval [CI]: 0.693–0.710) and 0.699 (95% CI: 0.682–0.716), respectively. The preoperative nomogram incorporated age, tumor size, location, grade, depth of invasion, regional lymph node(s) status, and metastatic site. The c-indices for the internal (bootstrap) and external validation sets were 0.629 (95% CI: 0.620–0.639) and 0.607 (95% CI: 0.588–0.626), respectively. Based on the preoperative nomogram, patients with preoperative total score > 28 showed no survival benefit with gastrectomy compared to no primary tumor surgery.

**Conclusions:**

Our survival nomograms for patients with metastatic gastric adenocarcinoma undergoing palliative gastrectomy can assist surgeons in treatment decision-making and prognostication.

## Background

Gastric cancer is one of the leading causes of cancer-related mortality worldwide [1]. Adenocarcinoma accounts for the majority of gastric cancer diagnoses, and patients with gastric adenocarcinoma often experience relatively short survival times. Surgery provides a curative opportunity for a number of patients and is considered the foundation of multimodal management of gastric cancer. However, a substantial proportion of gastric adenocarcinomas are advanced or metastatic disease at the time of diagnosis. Surgery is generally not a priority recommendation in such circumstances, except for patients with potentially life-threatening complications such as gastrointestinal bleeding, perforation, or obstruction.

A randomized clinical trial REGATTA which aimed to evaluate whether the addition of gastrectomy to chemotherapy improves survival for advanced gastric cancer patients with a single non-curable factor was terminated ahead of time due to the negative results from the interim analysis [2]. However, retrospective studies have produced evidence of a potential benefit to palliative gastrectomy in patients with stage IV gastric cancer [3–8]. A previous systematic review and meta-analysis of 14 studies comprising 3003 patients with incurable advanced gastric cancer revealed that palliative gastrectomy significantly improved overall survival [9]. Moreover, a recent study based on the Surveillance, Epidemiology, and End Results Program (SEER) data demonstrated a survival benefit for palliative gastrectomy in gastric cancer patients with stage IV disease after balancing baseline characteristics using propensity score matching analysis [10]. Results derived from non-consecutive studies demonstrated that patients who were younger, had better preoperative nutritional status, exhibited less nodal involvement, and underwent postoperative chemotherapy often experienced better outcomes [8]. Palliative primary tumor resection also offers advantages to patients with normal levels of serum carcinoembryonic antigen and/or normal CA19–9 [7], as well as to those with metastases confined to a single site [3].

However, the decision regarding whether to perform primary tumor surgery remains challenging, and investigations into models that are predictive of prognosis are scarce. As such, there is a need to identify and appraise factors that predict the outcomes of palliative gastrectomy in patients with metastases at the time of initial diagnosis of gastric adenocarcinoma, either for predicting the individuals’ prognoses or for clinical decision-making. Therefore, we performed this study using the SEER database to construct nomograms that predict the survival of patients with metastatic gastric adenocarcinoma subsequent to palliative gastrectomy.

## Methods

### Database and patient selection

**Fig. 1:**
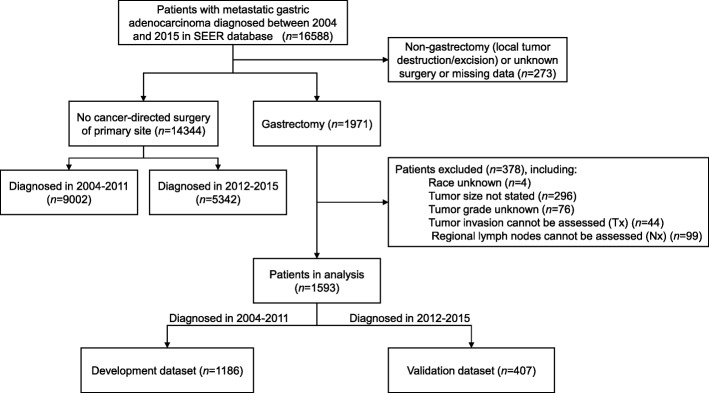
Flowchart of data selection. SEER, Surveillance, Epidemiology, and End Results Program

The recently released SEER database submission [Incidence - SEER 18 Regs Custom Data (with additional treatment fields), Nov 2017 Sub] includes cancer patients diagnosed from 1973 to 2015. We accessed the database using the SEER*Stat software version 8.3.5 (National Cancer Institute, USA) with permission from the SEER program office. There were 16,588 patients with metastatic gastric adenocarcinoma (International Classification of Diseases for Oncology-3 histologic type/behavior code: 8140/3–8389/3) in the database who were diagnosed between 2004 and 2015. Of these patients, 1971 were known to have been undergone a gastrectomy and 14,344 had not had surgery on the primary tumor. After excluding patients with missing essential data, 1593 patients with advanced gastric adenocarcinoma (i.e., with distant metastases at presentation) who received palliative gastrectomy were analyzed; 1186 patients (approximately three-quarters of the dataset) who were diagnosed between 2004 and 2011 were used as the development cohort to construct predictive models, while the remaining 407 patients (who were diagnosed between 2012 and 2015) were used as the validation cohort. Figure 1 shows the flowchart of data selection.

### Recoding and transformation of variables

Some variables in the original dataset were re-coded for purposes of statistical analysis. Tumors located in the fundus and body of the stomach, gastric antrum, pylorus, and lesser or greater curvature of stomach were re-classified as non-cardia tumors. Consequently, the primary lesion sites were categorized as “cardia tumors”, “non-cardia tumors”, “overlapping lesions”, and “stomach, not otherwise specified (NOS)”. Because relatively few patients had well-differentiated (grade [G]1) and undifferentiated (G4) tumors, they were combined with patients with moderately differentiated tumors (G2) and poorly differentiated tumors (G3), respectively, and the tumor grades were thus re-classified as G1/2 and G3/4. Because patient records spanned more than 10 years, staging according to both the American Joint Committee on Cancer (AJCC) sixth and seventh editions coexisted in the dataset; therefore, we translated the AJCC sixth edition T and N staging codes into their corresponding seventh edition codes to generate a uniform dataset.

Three continuous variables, “age at diagnosis”, “tumor size”, and “number of regional lymph nodes examined” were transformed into categorical variables. The X-tile plotting software was used to determine the cut-off value for continuous variables in terms of their impact on survival [11]. The analysis of the development dataset revealed that patients with more than 10 lymph nodes examined during surgery had significantly superior survival rates compared to those who had fewer than 10 lymph nodes examined. Similarly, survival curves were also separated into three age groups divided by two cut-off points: 65 and 80 years, as well as three prognostic groups by two tumor size cut-off points: 30 and 50 mm. Therefore, in the final Cox proportional hazards models and nomograms, tumor size was presented as three subgroups: “≤30 mm”, “31-50 mm”, and “> 50 mm”. Age was presented as “≤65 years”, “66–80 years”, and “> 80 years”. The number of regional lymph nodes examined was divided into the “≤10” and “> 10” groups.

### Construction of nomograms

The development dataset (i.e., patients diagnosed between 2004 and 2011) was used to construct the nomograms. Parameters that were patient-related (age, sex, and race), tumor-related (tumor size, location, pathological subtype, grade, T stage, N stage, and metastatic site), and treatment-related (scope of gastrectomy, examined lymph nodes, surgery to other sites, chemotherapy, and radiotherapy) were included in the Cox regression analysis. Cox regression was performed using SPSS 22.0 statistical software (IBM Corp., Armonk, NY). The backward stepwise method, including parameters with *P*-values < 0.01 and excluding those with P-values > 0.10, was used in combination with multivariate Cox proportional hazards regression analysis to select prognosis predictive variables for the nomogram. The results were described as hazards ratios (HRs) and 95% confidence intervals (CIs). All *P*-values were 2-tailed, and values of less than 0.05 were considered statistically significant.

We hypothesized that survival can be predicted preoperatively in patients who underwent palliative gastrectomy; hence, a preoperative model was constructed. This model incorporated “age at diagnosis”, “sex”, “race”, “grade”, “tumor size”, “tumor location”, “pathological subtype”, “tumor invasion” (according to T stage), “regional LN(s) status” (negative or positive, according to N stage) and “metastatic sites” into Cox regression analyses. It was assumed that these parameters could be evaluable pre-surgically. Although “T stage” and “N stage” are generally derived from postoperative pathological findings, modern imaging techniques can produce accurate staging algorithms, at least for negative or positive regional lymph node (LN) status.

Two nomograms were devised based on the above Cox models by scoring the independent variables according to the regression coefficient. Nomograms were plotted using the “nomogram” function in ‘R’ version 3.5.0 (The R Foundation for Statistical Computing, Vienna, Austria) using the ‘rms’ and ‘survival packages’ (http://www.r-project.org/).

### Validation of nomograms

The accuracies of the nomogram models were assessed using discrimination and calibration. Discrimination is the ability of the models to separate patients according to their survival status, and was measured using the Harrell concordance index (c-index). Similar to the area under the receiver operating characteristic curve, the c-index estimates the probability of concordance between predicted and observed outcomes in rank order and ranges between 0.5 and 1.0; higher values indicate better discrimination. Calibration refers to the discrepancy between predictions and actual outcomes, and is usually measured by graphic calibration curves that represent the relationship between the observed outcome frequencies and predicted probabilities.

The validation procedures were also performed using ‘R’ version 3.5.0. For internal validation of predictive models, 1000 bootstraps with sample sizes of 200 were generated from the original dataset. We calculated c-indices and plot calibration curves to compare the nomogram-predicted 1-year and 2-year survival probabilities with the actually-observed survival outcomes. Plots that approached a 45-degree line indicate a well-calibrated nomogram. The external validation dataset comprised patients diagnosed during a different period (2012–2015), and 1000 times bootstrapping (size 130) was performed to calculate and plot the calibration curves.

## Results

### Characteristics of patients in the datasets

The development dataset comprised 1186 patients diagnosed between 2004 and 2011, while the validation dataset included 407 patients diagnosed between 2012 and 2015. Table 1 shows the demographic, clinical, pathological, and treatment-related characteristics of the patients in this study. Patients older than 65 years accounted for 56.2% of the development cohort, with a median age 68 years old; 66.2% were male and 64.3% were Caucasian-Americans. Intestinal and diffuse subtype accounted for 18.5 and 8.9%, 72.6% patients were other adenocarcinoma or NOS. Most tumors (875 or 73.8%) were poorly differentiated (G3) or undifferentiated (G4). More than half (56.7%) of the primary tumors in the stomach penetrated the serosa (T4a) or invaded adjacent structures (T4b). Pure distant lymph node metastasis were found in 208 patients (17.5%), while 839 (70.7%) had synchronous visceral metastasis (i.e., distant metastases other than positive distant lymph nodes, carcinomatosis, Krukenberg tumors [metastases to the ovaries], or malignant ascites). Only 278 patients (23.4%) received total or near-total gastrectomy. The median number of examined regional lymph nodes was 12, with metastases noted in 1044 patients (88.0%). The characteristics of the patients in the validation cohort were largely similar to those of patients in the development cohort.

**Table 1:** Demographic, clinical, pathological, and surgical characteristics of the patients

Variables	Total population [*n* (%), *n* = 1593]	Development set [*n* (%), *n* = 1186]	Validation set [*n* (%), *n* = 407]
Age [median (IQR^a^)]	67.0 (57.0, 77.0)	68.0 (57.0, 77.0)	66.0 (55.0, 77.0)
≤65	715 (44.9)	520 (43.8)	195 (47.9)
66–80	644 (40.4)	492 (41.5)	152 (37.3)
> 80	234 (14.7)	174 (14.7)	60 (14.7)
Sex			
Male	1032 (64.8)	785 (66.2)	247 (60.7)
Female	561 (35.2)	401 (33.8)	160 (39.3)
Race			
White	1014 (63.7)	763 (64.3)	251 (61.7)
Black	240 (15.1)	181 (15.3)	59 (14.5)
Other	339 (21.3)	242 (20.4)	97 (23.8)
Tumor location			
Cardia	269 (16.9)	213 (18.0)	56 (13.8)
Non-cardia	998 (62.6)	738 (62.2)	260 (63.9)
Overlapping lesion	182 (11.4)	128 (10.8)	54 (13.3)
Stomach, NOS^b^	144 (9.0)	107 (9.0)	37 (9.1)
Tumor size (mm) [median (IQR)]	55.0 (40.0, 80.0)	55.0 (40.0, 80.0)	58.0 (40.0, 80.0)
≤30	230 (14.4)	172 (14.5)	58 (14.3)
31–50	466 (29.3)	343 (28.9)	123 (30.2)
>50	897 (56.3)	671 (56.6)	226 (55.5)
Pathological subtype			
Intestinal type	317 (19.9)	219 (18.5)	98 (24.1)
Diffuse type	167 (10.5)	106 (8.9)	61 (15.0)
Adenocarcinoma, NOS	959 (60.2)	754 (63.6)	205 (50.4)
Other adenocarcinoma	150 (9.4)	107 (9.0)	43 (10.6)
Grade			
Grade1/2	425 (26.7)	311 (26.2)	114 (28.0)
Grade3/4	1168 (73.3)	875 (73.8)	293 (72.0)
T stage (AJCC^c^ 7th)			
T1	73 (4.6)	55 (4.6)	18 (4.4)
T2	79 (5.0)	49 (4.1)	30 (7.4)
T3	519 (32.6)	409 (34.5)	110 (27.0)
T4a	577 (36.2)	402 (33.9)	175 (43.0)
T4b	345 (21.7)	271 (22.8)	74 (18.2)
N stage (AJCC 7th)			
N0	202 (12.7)	142 (12.0)	60 (14.7)
N1	706 (44.3)	522 (44.0)	184 (45.2)
N2	458 (28.8)	349 (29.4)	109 (26.8)
N3	227 (14.2)	173 (14.6)	54 (13.3)
Metastatic site(s)			
Distant LN(s)^d^	276 (17.3)	208 (17.5)	68 (16.7)
Viscera	1121 (70.4)	839 (70.7)	282 (69.3)
Viscera plus distant LN(s)	118 (7.4)	96 (8.1)	22 (5.4)
Distant metastasis, NOS	78 (4.9)	43 (3.6)	35 (8.6)
Gastrectomy			
Partial gastrectomy	808 (50.7)	612 (51.6)	196 (48.2)
Near-total or total gastrectomy	361 (22.7)	278 (23.4)	83 (20.4)
Gastrectomy, NOS	424 (26.6)	296 (25.0)	128 (31.4)
No. of regional LN(s) examined [median (IQR)]	13 (6, 21)	12 (6, 20)	14 (7, 22)
≤10	660 (41.4)	518 (43.7)	142 (34.9)
>10	933 (58.6)	668 (56.3)	265 (65.1)
Surgery to other site(s) or node(s)			
Yes	404 (25.4)	309 (26.1)	95 (23.3)
No	1189 (74.6)	877 (73.9)	312 (76.7)
Chemotherapy			
Yes	802 (50.3)	579 (48.8)	223 (54.8)
No/Unknown	791 (49.7)	607 (51.2)	184 (45.2)
Radiotherapy			
Yes	254 (15.9)	192 (16.2)	62 (15.2)
No/Unknown	1339 (84.1)	994 (83.8)	345 (84.8)
Sequence of radiotherapy with surgery			
Pre-operative radiotherapy	69/254 (27.2)	37/192 (19.3)	32/62 (51.6)
Post-operative radiotherapy	182/254 (71.7)	153/192 (79.7)	29/62 (46.8)
Intraoperative or sandwich	3/254 (1.2)	2/192 (1.0)	1/62 (1.6)

^a^interquartile range; ^b^not otherwise specified; ^c^American Joint Committee on Cancer; ^d^lymph node(s)

### Cox regression models

Fifteen parameters were initially incorporated the into Cox regression analyses, including three demographic variables, seven tumor-related variables, and five treatment-related variables (Postoperative Model); the analysis results are shown in Table 2. T and N staging were consistently significant prognostic factors. Except for T2 tumors with a statistically insignificant increased risk of death, the risk of death in patients with ≥T3 tumors was approximately 2-fold greater than in those with T1 tumors, with HRs of 1.9 (95% CI: 1.3–2.7), 2.3 (95% CI: 1.6–3.3), and 2.5 (95% CI: 1.7–3.5) for T3, T4a, and T4b tumors, respectively (all *P* < 0.05). The risk of death was also greater in patients with ≥N1 tumors than in those with N0 tumors; moreover, older age and poorer tumor differentiation were also associated with poorer survival. However, patients with only distant lymph node involvement had a significantly lower risk of death than patients with metastases at other sites; non-cardia tumors showed a lower risk of death than cardia tumors. Chemotherapy and radiotherapy significantly decreased the risk of death with HRs of 0.5 (95% CI: 0.4–0.6) and 0.8 (95% CI: 0.6–0.9). Sex, race, pathological subtype and surgery to other site(s) or node(s) did not have a significant impact on survival. Tumor size > 50 mm was associated with a marginally decreased risk of death compared to the “≤30 mm” group (HR: 0.8, 95%CI: 0.7–1.0, *P* = 0.069). Based on the backward stepwise method, “age”, “tumor size”, “tumor location”, “grade”, “T stage”, “N stage”, “metastatic site”, “scope of gastrectomy”, “number of examined lymph node(s)”, “chemotherapy” and “radiotherapy” were retained in the final regression equation.

**Table 2:** Multivariate Cox proportional hazards regression analysis of the development cohort

	Postoperative Model			Preoperative Model		
	*HR* ^a^	*95% CI* ^b^	*P*	*HR*	*95% CI*	*P*
Age (“≤65”as reference)			0.004			0.000
66–80	1.075	0.935–1.236	0.311	1.285	1.123–1.470	0.000
>80	1.391	1.143–1.694	0.001	1.977	1.643–2.378	0.000
Sex (“Male” as reference)						
Female	0.961	0.843–1.095	0.548	0.990	0.869–1.129	0.885
Race (“White” as reference)			0.600			0.593
Black	0.984	0.823–1.177	0.863	0.962	0.805–1.148	0.665
Other	0.923	0.789–1.079	0.313	0.923	0.791–1.078	0.314
Tumor location (“Cardia” as reference)			0.008			0.020
Non-cardia	0.816	0.684–0.974	0.024	0.935	0.785–1.113	0.449
Overlapping lesion	1.054	0.829–1.340	0.669	1.241	0.977–1.577	0.077
Stomach, NOS^c^	1.014	0.784–1.312	0.915	1.129	0.876–1.455	0.348
Tumor size (“≤30 mm” as reference)			0.033			0.041
31-50 mm	0.985	0.802–1.209	0.882	1.120	0.914–1.373	0.273
> 50 mm	0.833	0.684–1.014	0.069	0.938	0.773–1.138	0.515
Subtype (“Intestinal type” as reference)			0.536			0.267
Diffuse type	0.861	0.664–1.117	0.260	0.925	0.717–1.193	0.547
Adenocarcinoma, NOS	0.997	0.846–1.175	0.972	1.095	0.932–1.287	0.269
Other adenocarcinoma	0.906	0.708–1.159	0.431	0.943	0.738–1.206	0.642
Grade (“Grade1/2” as reference)						
Grade3/4	1.334	1.155–1.540	0.000	1.269	1.100–1.463	0.001
T category-AJCC^d^ 7th (“T1” as reference)			0.000			0.000
T2	1.395	0.894–2.179	0.143	1.252	0.803–1.953	0.321
T3	1.906	1.341–2.708	0.000	1.658	1.171–2.348	0.004
T4a	2.284	1.594–3.273	0.000	1.971	1.382–2.811	0.000
T4b	2.457	1.704–3.542	0.000	2.128	1.481–3.058	0.000
N category-AJCC 7th (“N0” as reference)			0.000			
N1	1.341	1.082–1.662	0.007	–	–	–
N2	2.003	1.579–2.542	0.000	–	–	–
N3	2.128	1.617–2.801	0.000	–	–	–
N+	–	–	–	1.397	1.137–1.717	0.001
Metastatic site(s) (“Distant LN(s)^e^” as reference)			0.000			0.000
Viscera	1.496	1.255–1.783	0.000	1.586	1.334–1.886	0.000
Viscera plus distant LN(s)	1.810	1.389–2.359	0.000	1.782	1.372–2.314	0.000
Distant metastasis, NOS	1.748	1.223–2.497	0.002	1.664	1.173–2.362	0.004
Gastrectomy (“Partial gastrectomy” as reference)			0.078			
Near-total or total gastrectomy	0.838	0.718–0.978	0.025	–	–	–
Gastrectomy, NOS	0.930	0.801–1.080	0.343	–	–	–
No. of regional LN(s) examined (“≤10” as reference)						
> 10	0.684	0.591–0.792	0.000	–	–	–
Surgery to other site(s) or node(s) (“No” as reference						
Yes	1.008	0.877–1.159	0.913	–	–	–
Chemotherapy (“No/Unknown” as reference						
Yes	0.514	0.448–0.589	0.000	–	–	–
Radiotherapy (“No/Unknown” as reference						
Yes	0.778	0.647–0.936	0.008	–	–	–

^a^hazard ratio; ^b^confidence interval; ^c^not otherwise specified; ^d^American Joint Committee on Cancer; ^e^lymph node(s)

In the preoperative Cox regression model, older age, poorer differentiation, deeper tumor invasion, and positive regional LN(s) were shown to increase the risk of death significantly. Pure distant LN(s) metastasis had a lower risk of death than metastases in other site(s). After performing the backward stepwise procedure, the above variables together with tumor location and tumor size were retained in the final regression equation. The results were also shown in Table 2.

### Postoperative nomogram and validation

**Fig. 2:**
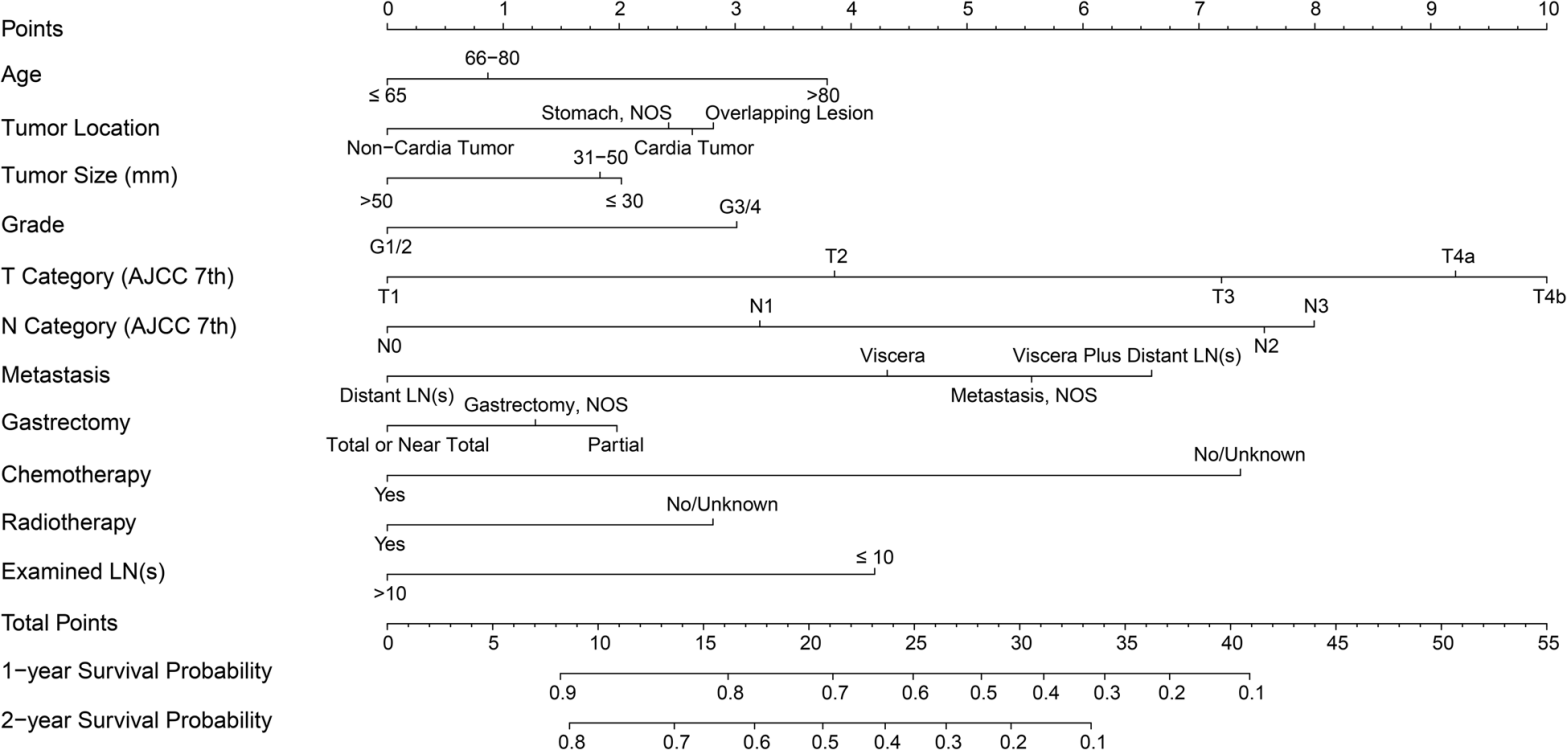
Postoperative prognostic nomogram for patients with metastatic gastric adenocarcinoma who undergo palliative gastrectomy. Visceral metastasis is defined as distant metastasis other than positive distant lymph nodes, carcinomatosis, Krukenberg tumors (metastases to the ovaries), or malignant (tumor cell-positive) ascites. AJCC, American Joint Committee on Cancer; NOS, not otherwise specified; LN, lymph node

**Fig. 3:**
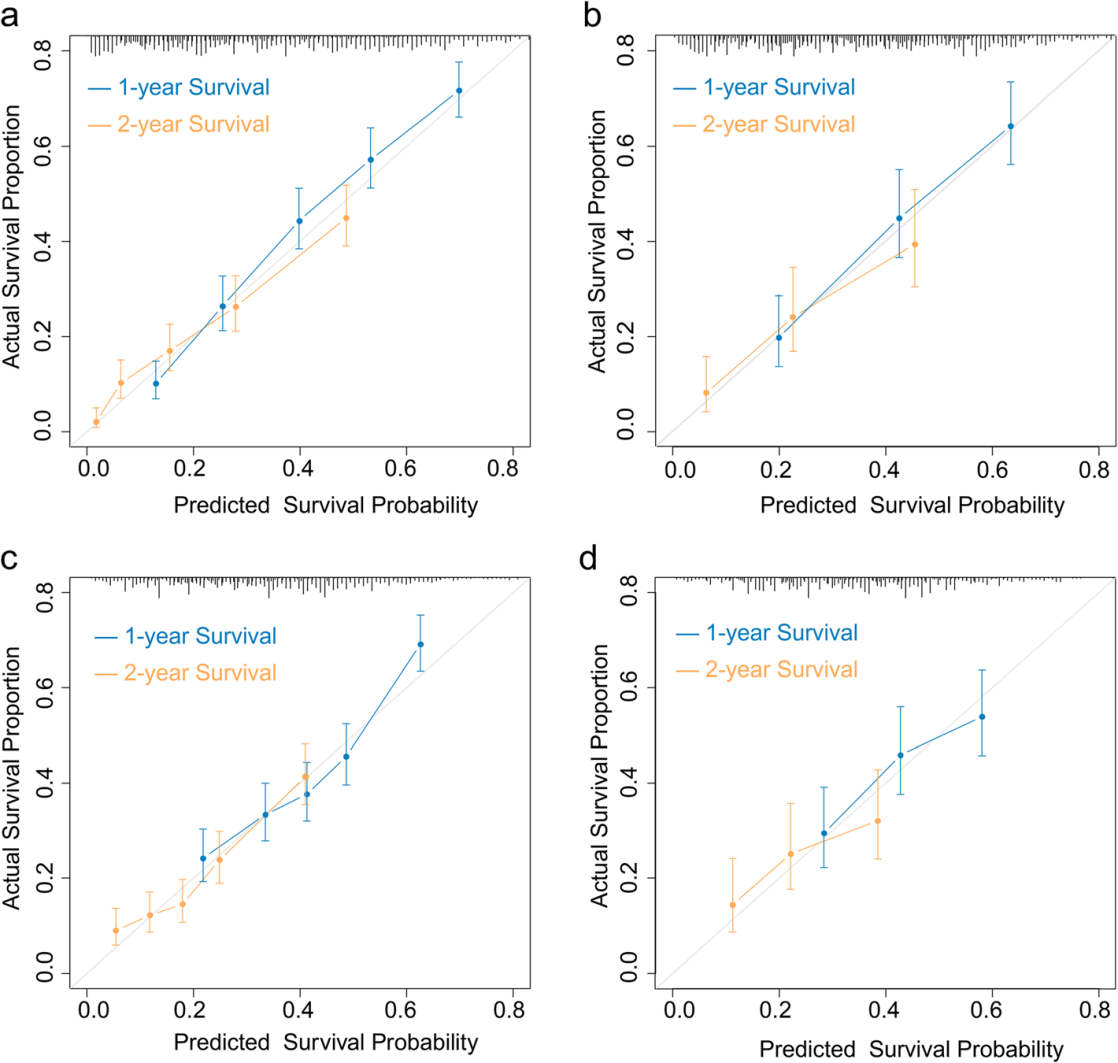
Calibration curves for the nomograms for patients with metastatic gastric adenocarcinoma who undergo palliative gastrectomy. **a**: Bootstrap validation of postoperative prognostic nomogram with samples sizes of 200 in the development dataset. **b**: External validation of postoperative prognostic nomogram using the cohort of 407 patients with samples sizes of 130. **c**: Bootstrap validation of preoperative nomogram with samples size of 200 in the development dataset. **d**: External validation of preoperative nomogram using the 407-patient validation cohort. The 45-degree grey lines show the ideal reference lines where the predicted survival probabilities match the actual survival proportions. Dots indicate the predicted probabilities for the resampled groups of patients with their respective 95% confidence intervals

After running the R program, we plotted a nomogram for the determination of postoperative prognosis (Fig. 2). Eleven parameters were scored using various points within a scale of 1 to 10 as follows: age at diagnosis (0 for age ≤ 65 years, 0.9 for age 66–80 years, and 3.8 for age > 80 years), tumor location (0 for non-cardia tumor, 2.6 for cardia tumor, 2.8 for overlapping lesion, and 2.4 for NOS), tumor size (0 for > 50 mm, 2.0 for ≤30 mm, and 1.8 for 31-50 mm), grade (0 for grade 1/2 and 3.0 for grade 3/4), T invasion (0, 3.9, 7.2, 9.2, and 10.0 for T1, T2, T3, T4a, and T4b, respectively), N group (0, 3.2, 7.6, and 8.0 for N0, N1, N2, and N3, respectively), metastatic site (0, 4.3, 6.6, and 5.6 for “distant lymph node metastasis”, “visceral metastasis”, “visceral plus distant lymph node metastasis”, and “distant metastasis, NOS”, respectively), the number of examined regional lymph nodes (0 for > 10 and 4.2 for ≤10), scope of gastrectomy (0 for total or near total, 2.0 for partial, and 1.3 for gastrectomy, NOS), chemotherapy (0 for yes, 7.4 for no/unknown), and radiotherapy (0 for yes, 2.8 for no/unknown). The total above scores were summed to calculate the individuals’ 1-year and 2-year survival rates. The c-index for the postoperative nomogram model obtained by bootstrap resampling was 0.701 (95% CI: 0.693–0.710). Bootstrap validation was performed to plot the nomogram-predicted 1-year and 2-year survival probabilities against the corresponding observed survival rates obtained by the Kaplan-Meier method; the calibration curves for the postoperative model are shown in Fig. 3a. When applying the postoperative nomogram to the validation dataset, the c-index was 0.699 (95% CI: 0.682–0.716); the calibration curves are illustrated in Fig. 3b. The results indicated moderate discrimination and good calibration for the model in the validation cohort.

### Preoperative nomogram and validation

**Fig. 4:**
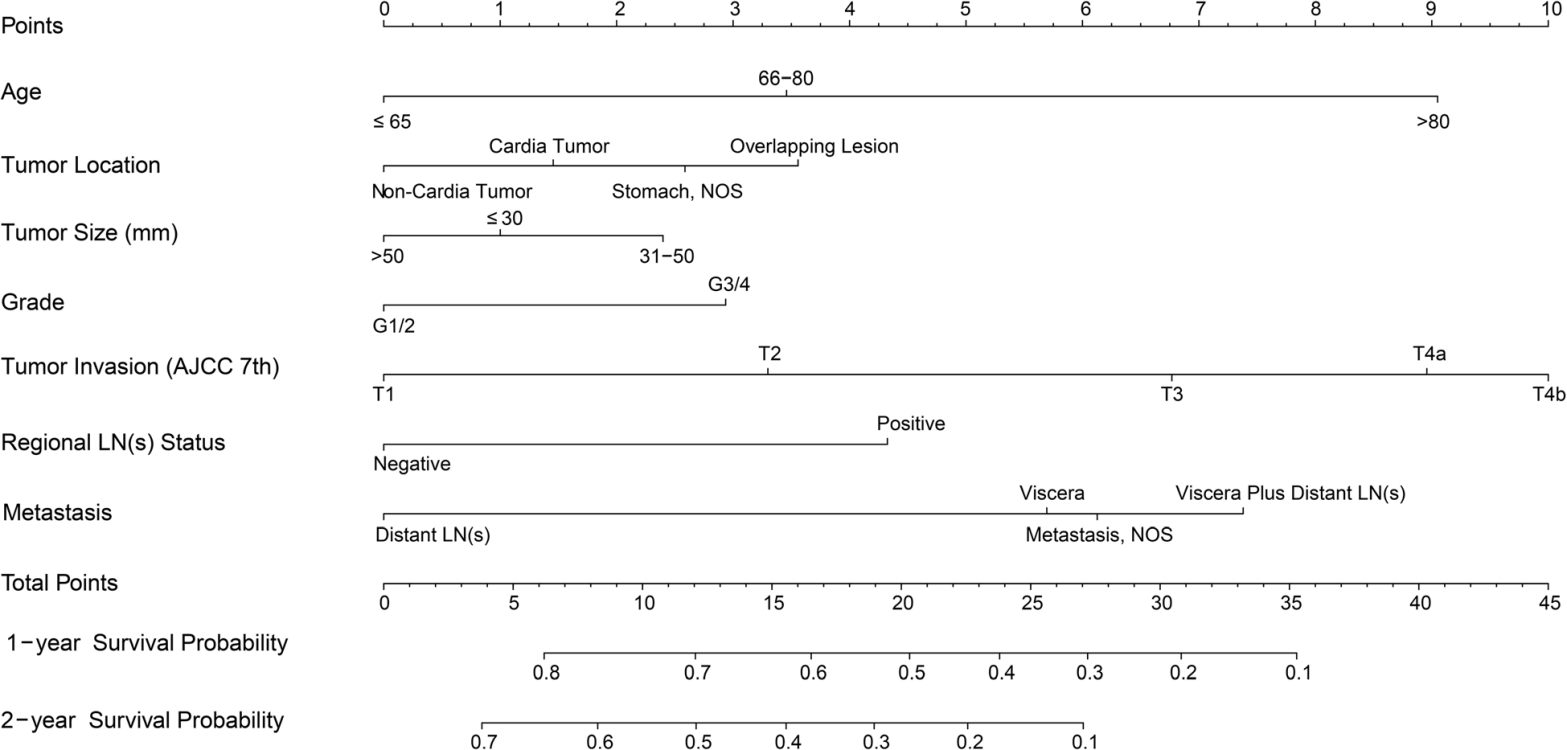
Preoperative prognostic nomogram for metastatic gastric adenocarcinoma patients who undergo gastrectomy. Visceral metastasis is defined as distant metastasis other than positive distant lymph nodes, carcinomatosis, Krukenberg tumors (metastases to the ovaries), or malignant (tumor cell-positive) ascites. AJCC, American Joint Committee on Cancer; NOS, not otherwise specified; LN, lymph node

A similar procedure to the above was performed to generate a preoperative nomogram based on a preoperative model (Fig. 4), which was designed to predict prognoses before making surgical decisions. Seven preoperatively measurable predictors and assigned scores of the values were as following: age ≤ 65, 66–80, and ≥ 80 years were scored as 0, 3.5, and 9.1 points, respectively; non-cardia tumor was scored as 0, while cardia tumor, overlapping lesion and tumor, NOS were scored as 1.5, 3.6, 2.6 respectively; tumor > 50 mm was scored as 0, ≤30 mm was scored as 1.0 and 31-50 mm was scored as 2.4; grades 1/2 and 3/4 were scored as 0 and 2.9 points; “T1”, “T2”, “T3”, “T4a”, and “T4b” were scored as 0, 3.3, 6.8, 9.0, and 10.0 points, respectively; negative regional LN(s) and positive regional LN(s) was scored as 0 and 4.3. As for sites of metastasis, “distant lymph node-only metastasis” was scored as 0, “visceral metastasis” as 5.7, “visceral plus distant lymph node metastasis” as 7.4, and “distant metastasis, NOS” as 6.1. The c-index of the postoperative nomogram using bootstrap and external validation were 0.629 (95% CI: 0.620–0.639) and 0.607 (95% CI: 0.588–0.626) respectively. Figure 3c and d shows the calibration curves of the predicted 1-year and 2-year survival probabilities versus actual survival rates based on bootstrap resampling and validation data.

### Determination of cut-off value for preoperative nomogram score

**Fig. 5:**
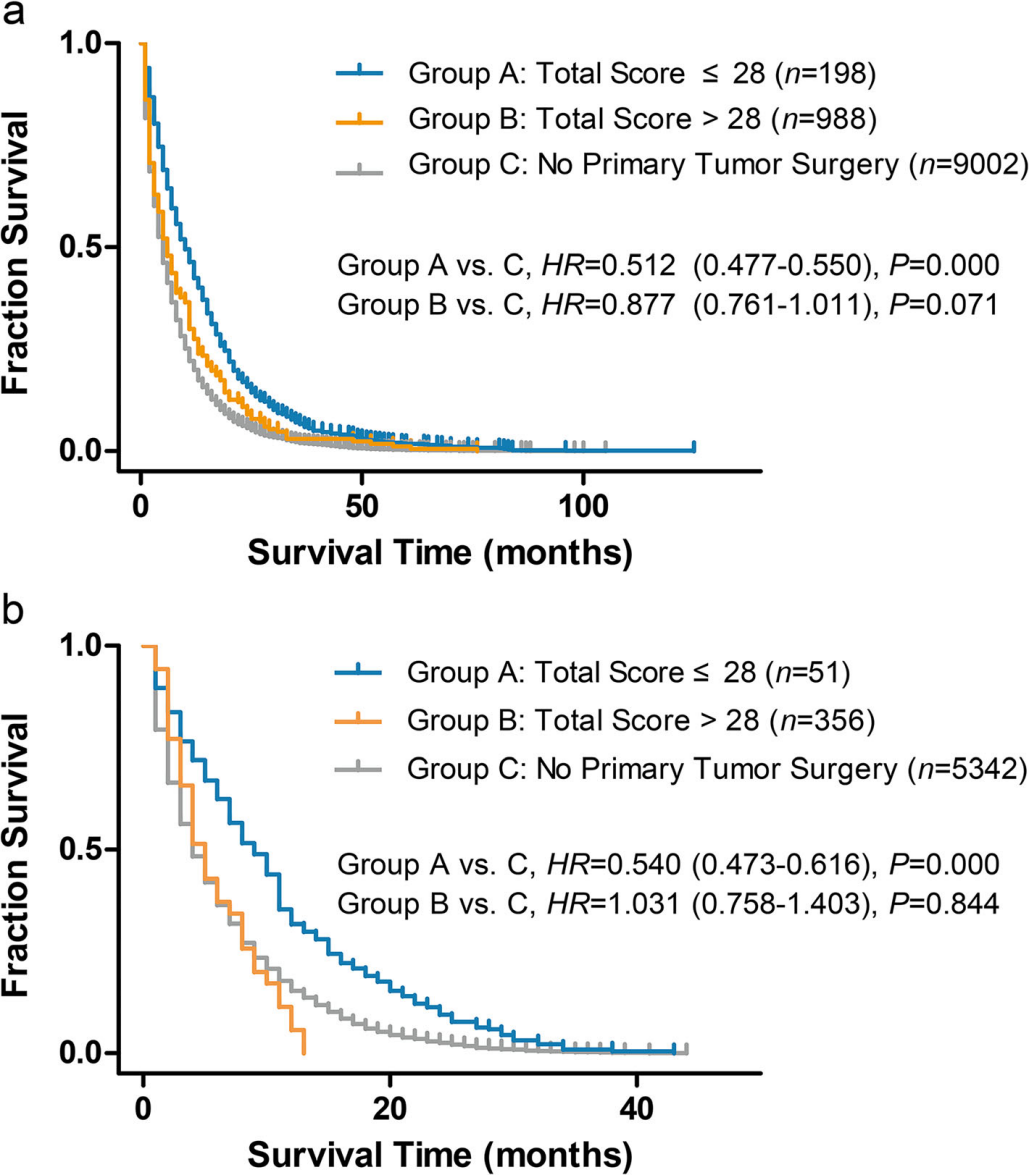
Survival curves for metastatic gastric adenocarcinoma patients who undergo gastrectomy and those without primary tumor surgery. Total scores were calculated according to preoperative nomogram. **a**: Total score cut-off value of 28 was set in development cohort, Cox proportional hazards regression was used to compare their survival with contemporarily registered cases who without primary tumor resection. **b**: Patients in validation cohort were divided into two groups by using a total score cut-off value of 28, Cox proportional hazards regression was used to compare their survival with contemporarily registered cases who without primary tumor resection

Cases in the development dataset were scored base on preoperative nomogram, and total scores were calculated. A cut-off value was attempted several times manually by comparing survival with that of contemporarily registered cases with no primary tumor surgery. A total score of 28 was found to be an optimal cut-off point. After setting the “no primary tumor surgery group” as reference in the Cox regression, there was no survival benefit for patients with a total score > 28 (HR = 0.877, 95% CI: 0.761–1.011, *P* = 0.071). In fact, survival for patients with a total score ≤ 28 was shown to be superior to the “no primary tumor surgery group” (HR = 0.512, 95%CI: 0.477–0.550, *P* = 0.000); survival curves are showed in Fig. 5a. We then applied the scoring system in the preoperative nomogram to the validation dataset. Patients in the validation dataset were divided into two groups based on a total score ≤ 28 and a total score > 28, while comparing survival to that of the “no primary tumor surgery group” (contemporarily registered). The group with a total score > 28 showed no survival benefit [median overall survival (mOS): 5.0 (3.0–7.0) vs. 5.0 (4.7–5.3) months, HR: 1.031(95% CI: 0.758–1.403), *P* = 0.844). Those with a total score ≤ 28 group showed improved survival (mOS: 12.0 (10.1–13.8) vs. 5.0 (4.7–5.3) months, HR: 0.540 (95% CI: 0.473–0.616), *P* = 0.000); survival curves are showed in Fig. 5b.

## Discussion

A staging-guided treatment strategy is the mainstay of clinical management for the vast majority of cancer patients. Evidence exists for supporting primary site surgery in patients with various stage IV cancers such as renal cancer [12, 13], breast cancer [14, 15], and colorectal cancer [16, 17]. It was suggested that resection of the primary tumor could potentially reduce the immunosuppressive tumor burden or remove the source of new metastases [18]. Although the REGATTA study failed to demonstrate survival benefit of palliative gastrectomy plus chemotherapy in advanced gastric cancer patients [2]. Results of retrospective SEER database analysis prone to benefit of palliative gastrectomy [10]. In fact, in REGATTA study, about one-third tumors located in upper third of stomach in gastrectomy plus chemotherapy group, while cardia tumor accounted for only 16.9% in current SEER dataset. This may explain the disappointment results of REGATTA. We considered that selected patients with advanced gastric adenocarcinoma could benefit from palliative gastrectomy. We therefore developed prognostic nomograms aimed at identifying patients who would benefit from this procedure; our models will be helpful for individuals’ risk determination and clinical decision-making.

Most of the studies mentioned above that demonstrated survival benefit of palliative gastrectomy were conducted in Asian countries, but given the paucity of cases of metastatic gastric cancer with palliative gastrectomy in clinical practices, a retrospective analysis in single center is difficult to conduct. In addition, analysis based on the SEER data demonstrated a survival benefit for palliative gastrectomy in stage IV gastric cancer patients [10]. We selected the SEER database, which included only American cases, in current study. And the results of this analysis are not necessarily transferable to Asian patients.

The postoperative nomogram provided individualized estimates of overall survival for patients with metastatic gastric adenocarcinoma who received palliative gastrectomy. Similar to lymph node dissection for operable diseases [19], this nomogram as well as the findings of another study [20] showed that regional lymph node dissection during palliative surgery has a marked impact on survival. Therefore, we strongly recommend extensive lymph node dissection, such as D2 lymphadenectomy, even during palliative gastrectomy.

Other than patients who develop emergency complications, surgeons occasionally face a dilemma regarding whether to perform gastrectomy on gastric cancer patients with metastases present at the time of initial diagnosis, especially those with supposedly more favorable prognostic factors, such as good performance status or single/oligometastases. Our preoperative nomogram should be useful in such circumstances. Although there remains no consensus regarding preoperative T and N staging for gastric cancer, we hold that evolving imaging modalities such as abdominal multi-detector row computed tomography (with isotropic or 3-dimensional imaging) [21], 3-Tesla magnetic resonance imaging [22], endoscopic ultrasonography with aspiration cytology [23, 24], or their combinations are likely to provide increasingly precise estimation for tumor invasion and lymph node status. By using some preoperatively measurable variables, the preoperative nomogram could, to a limited extent, help surgeons to make an appropriate decision, since patients with total scores more than 28 showed no benefit from palliative gastrectomy.

There are some limitations associated with nomograms such as ours. First, the validation cohort had similar characteristics to the development cohort, which restricted the extrapolation to more diverse patient groups. Ideally, such nomograms should be validated in an external cohort or using data from other institutions. Second, our nomograms showed moderate predictive capabilities, especially for the preoperative nomogram with c-indices no greater than 0.7. One possible explanation was that the parameters used to construct the models were limited to those available in the database. The database cannot provide more details of the patients, for example, performance status of the patients, oliogo- or multi-metastasis of tumor, et al.; as to the information on treatment, only sequence of radiotherapy with surgery was displayed in database, other details such as residues of tumor resection (R0, R1 or R2), chemotherapy sequence and concurrent chemo-radiation were omitted in current cohort. All these factors were considered to be have impact on survival of patients who received palliative gastrectomy. Finally, constructing the preoperative nomogram depended in part on postoperative parameters such as T and N staging as surrogates; if more relevant preoperative parameters been available, they would likely have been more valuable.

## Conclusions

We constructed first-of-their-type nomograms that are predictive of survival in patients with metastatic gastric adenocarcinoma who are candidates for palliative gastrectomy. Despite some limitations, these nomograms may assist surgeons in their decision-making regarding treatment as well as in evaluating their patients’ prognoses.

## Data Availability

The data that support the findings of this study are available from SEER program of the National Cancer Institute. However, restrictions apply to the availability of these data, which were used under license for the current study, and so are not publicly available. Data are, however, available from the authors upon reasonable request and with permission of SEER program office.

## Abbreviations

AJCC: American Joint Committee on Cancer
c-index: concordance index
CIs: Confidence intervals
G: Grade
HRs: Hazards ratios
mOS: median overall survival
NOS: Not otherwise specified
SEER: Surveillance, Epidemiology, and End Results Program

## References

[1] Van Cutsem E, Sagaert X, Topal B, Haustermans K, Prenen H (2016). Gastric cancer. Lancet..

[2] Fujitani K, Yang HK, Mizusawa J, Kim YW, Terashima M, Han SU (2016). Gastrectomy plus chemotherapy versus chemotherapy alone for advanced gastric cancer with a single non-curable factor (REGATTA): a phase 3, randomised controlled trial. Lancet Oncol.

[3] Chang YR, Han DS, Kong SH, Lee HJ, Kim SH, Kim WH (2012). The value of palliative gastrectomy in gastric cancer with distant metastasis. Ann Surg Oncol.

[4] Zhang JZ, Lu HS, Huang CM, Wu XY, Wang C, Guan GX (2011). Outcome of palliative total gastrectomy for stage IV proximal gastric cancer. Am J Surg.

[5] Kulig P, Sierzega M, Kowalczyk T, Kolodziejczyk P, Kulig J (2012). Non-curative gastrectomy for metastatic gastric cancer: rationale and long-term outcome in multicenter settings. Eur J Surg Oncol.

[6] Yazici O, Ozdemir N, Duran AO, Menekse S, Sendur MA, Karaca H (2016). The effect of the gastrectomy on survival in patients with metastatic gastric cancer: a study of ASMO. Future Oncol.

[7] Chiu CF, Yang HR, Yang MD, Jeng LB, Yang TY (2016). Palliative gastrectomy prolongs survival of metastatic gastric Cancer patients with Normal preoperative CEA or CA19-9 values: a retrospective cohort study. Gastroenterol Res Pract.

[8] Hsu JT, Liao JA, Chuang HC, Chen TD, Chen TH, Kuo CJ (2017). Palliative gastrectomy is beneficial in selected cases of metastatic gastric cancer. BMC Palliat Care.

[9] Sun J, Song Y, Wang Z, Chen X, Gao P, Xu Y (2013). Clinical significance of palliative gastrectomy on the survival of patients with incurable advanced gastric cancer: a systematic review and meta-analysis. BMC Cancer.

[10] He X, Lai S, Su T, Liu Y, Ding Y, Quan S (2017). Survival benefits of gastrectomy in gastric cancer patients with stage IV: a population-based study. Oncotarget.

[11] Camp RL, Dolled-Filhart M, Rimm DL (2004). X-tile: a new bio-informatics tool for biomarker assessment and outcome-based cut-point optimization. Clin Cancer Res.

[12] Mickisch GH, Garin A, van Poppel H, de Prijck L, Sylvester R (2001). European organisation for Reasearch and treatment of Cancer genitourinary group. Radical nephrectomy plus interferon-alfa-based immunotherapy compared with interferon alfa alone in metastatic renal-cell carcinoma: a randomised trial. Lancet.

[13] Graham J, Heng DY (2018). Real-world evidence in metastatic renal cell carcinoma. Tumori..

[14] Harris E, Barry M, Kell MR (2013). Meta-analysis to determine if surgical resection of the primary tumour in the setting of stage IV breast cancer impacts on survival. Ann Surg Oncol.

[15] Lane WO, Thomas SM, Blitzblau RC, Plichta JK, Rosenberger LH, Fayanju OM (2019). Surgical resection of the primary tumor in women with De novo stage IV breast Cancer: contemporary practice patterns and survival analysis. Ann Surg.

[16] Ahmed S, Leis A, Fields A, Chandra-Kanthan S, Haider K, Alvi R (2014). Survival impact of surgical resection of primary tumor in patients with stage IV colorectal cancer: results from a large population-based cohort study. Cancer.

[17] Ahmed S, Fields A, Pahwa P, Chandra-Kanthan S, Zaidi A, Le D (2015). Surgical resection of primary tumor in asymptomatic or minimally symptomatic patients with stage IV colorectal Cancer: a Canadian Province experience. Clin Colorectal Cancer.

[18] Danna EA, Sinha P, Gilbert M, Clements VK, Pulaski BA, Ostrand-Rosenberg S (2004). Surgical removal of primary tumor reverses tumor-induced immunosuppression despite the presence of metastatic disease. Cancer Res.

[19] Naffouje SA, Salti GI (2017). Extensive lymph node dissection improves survival among American patients with gastric adenocarcinoma treated surgically: analysis of the National Cancer Database. J Gastric Cancer.

[20] Zhuo C, Ying M, Lin R, Wu X, Guan S, Yang C (2017). Negative lymph node count is a significant prognostic factor in patient with stage IV gastric cancer after palliative gastrectomy. Oncotarget.

[21] Wang M, Ye Y, Yang Q, Li J, Han C, Wang W (2015). Pre-operative lymph node status of gastric cancer evaluated by multidetector computed tomography. Int J Clin Exp Med.

[22] Joo I, Lee JM, Kim JH, Shin CI, Han JK, Choi BI (2015). Prospective comparison of 3T MRI with diffusion-weighted imaging and MDCT for the preoperative TNM staging of gastric cancer. J Magn Reson Imaging.

[23] Pei Q, Wang L, Pan J, Ling T, Lv Y, Zou X (2015). Endoscopic ultrasonography for staging depth of invasion in early gastric cancer: a meta-analysis. J Gastroenterol Hepatol.

[24] Mocellin S, Pasquali S (2015). Diagnostic accuracy of endoscopic ultrasonography (EUS) for the preoperative locoregional staging of primary gastric cancer. Cochrane Database Syst Rev.

